# Sex differences and athletic performance. Where do trans individuals fit into sports and athletics based on current research?

**DOI:** 10.3389/fspor.2023.1224476

**Published:** 2023-10-27

**Authors:** D. J. Oberlin

**Affiliations:** Department of Exercise Sciences and Recreation, City University of New York, Lehman College, Bronx, NY, United States

**Keywords:** transgender, sports, gender, genderqueer, performance

## Abstract

There are well known sex differences in parameters of physical fitness/performance due to changes occurring during sexual development. Thus, many sport and athletic events have regulations separating male and female participants. However, the inclusion or exclusion of transgender individuals in athletics has recently received outsized attention despite relatively few cases of transgender athletes. When determining which athletic gender category trans individuals should be permitted to compete in, it is important to understand the level of physical fitness/performance these individuals possess relative to their cisgender counterparts. Unfortunately, there are few studies investigating this topic, and several complications that confound this research. The current review seeks to discuss sex and gender as concepts, review sex differences in fitness/performance and how they develop, and then, consider how current evidence suggests that trans individuals compare to cis individuals. Finally, this review seeks to offer considerations for whether trans individuals should be excluded from sports and athletics, and how future research should proceed to better understand this marginalized population.

## 1. Introduction

While transgender individuals have likely always existed, they have recently received an outsized level of attention and scrutiny from governments and other regulatory bodies (1–5). This scrutiny spans multiple issues, one of which has been participation in sports and athletics (1, 2, 6, 7). Concerns have been raised for whether trans individuals should be allowed to compete in sports with cisgender individuals, suggesting they should not compete in the category that aligns with their gender identity or that they should participate in a separate category entirely (1, 3–6, 8–11). Addressing these concerns requires consideration of several concepts: sex and gender, how sports and athletics currently regulate participation by sex and gender, potential human right violations or discrimination coming from exclusionary approaches, what sex differences exist in athletics and how these develop and affect athletic performance, and whether trans individuals' participation in sports inherently leads to risks of either inequity or injury. This is not a systematic review, and makes no claim to “solve” this matter. The purpose of the current review is to better describe the modern landscape of how and why gender is used as criteria for inclusion/exclusion from sport and athletics, and to drive further considerations of how or whether transgender individuals should be excluded from these events. To achieve this, pubmed and google scholar were used to search for terms including: “trans”, “gender”, “transgender”, “sports”, “performance”, “exercise”, “athletics”. Articles were included if they met the following criteria: (1) original research rather than review, (2) research compared transgender individuals on gender affirming hormone therapy to cisgender individuals rather than comparing cis men and cis women, and (3) some measures of fitness or performance were measured. This was done to find articles that investigated transgender performance, accounting for hormonal differences, and comparing these with cisgender counterparts. Articles were excluded if no more than one trans individual was compared to cisgender individuals. These criteria were used for the following reasons. (1) Transgender individuals on gender affirming hormone therapy should not be equated to their pre-transition cisgender counter parts. Gender affirming hormone therapy can alter physiologic parameters (12–16). Thus, transgender individuals must be directly measured rather than assuming cisgender sex differences are analogous to cisgender and transgender differences. (2) To avoid assuming that a single transgender individual can be generalized to an entire population. While case studies can be informative, selecting an exceptional trans athlete or a sedentary trans individual and assuming they represent an entire community would likely lead to misleading conclusions.

## 2. Sex and gender

### 2.1. Distinguishing sex and gender

Before discussing gender as a criterion for sports participation, the concepts of sex and gender should be clearly understood. Although often used synonymously, sex and gender are two different terms used to describe related concepts. Sex is a biological concept having to do with chromosomes, genitalia, gonads, and hormones (17–19). While related to sex, gender has to do with behaviors, societal roles/expectations, and attributes which are valued or discouraged within a social group (19–21). These related concepts can be conflated, as biological sex and bodily appearance influences how members within a society interact with one another (20, 22). The various interactions among members of a community pressure individuals within the community to behave in a particular manner or display certain characteristics based on their perceived sex (20, 22). To a casual observer, the differences between sex and gender may seem trivial, however they address individual qualities which have wide variations even within what may be considered a single group. Thus, to further a discussion of transgender individuals in sport and athletics, the concepts of sex and gender must first be understood, although they may be impossible to entirely disentangle.

Prior to understanding chromosomes, sex was determined to be male or female based on the genitalia possessed by an individual and their secondary sex characteristics (23–25). However, by the early 20th century, scientists were beginning to understand that chromosomes shared between mother and father would determine something about the sex of the offspring, but it would still be decades before a greater understanding of the variety of possible outcomes could be better understood (26–29). Currently, chromosomes, gonads, hormones, and genitals (and to a lesser extent secondary sex characteristics) have all been used to describe the concept of biological sex in humans (18). While chromosomal varieties can certainly result in XY males and XX females, it is also possible for individuals to be born with sex chromosome aneuploidy leading to variations such as XXX, XXY, XYY, XXXX, XXYY, XXXY, XXXXY, or even XXXXX (30–34). Differences in sex chromosomes have also led to discoveries of sex chromosome dose effects leading to variations in gene regulation as well as in body size and other phenotypic differences (33, 34). Besides variations in chromosomes, there are other possible anatomical deviations from what is typically considered male or female, such as males with ambiguous external genitalia, a uterus, and fallopian tubes to females who also carry a Y chromosome, have ambiguous external genitalia, and possess both ovaries and testis (18, 35). Hormonal concentrations, particularly of testosterone, are also used to distinguish male from female (18, 36). Both males and females have some concentration of both testosterone and estrogen, and these concentrations vary among individuals (37–39). However, testosterone is frequently used as a marker to distinguish male from female as males have 15–20 × more testosterone than females (36–38, 40). Despite this, there are also wide variations within cisgender men and women which do not make them more or less male or female (36–38). Hence, even a biological concept of sex, whether based on chromosomes, anatomy, hormones, or other criteria, does not fit neatly into two rigid categories (17, 18).

Gender is a more complicated topic than biological sex, because gender, according to the World Health Organization, “refers to the characteristics of women, men, girls and boys that are socially constructed” (20–22). Due to being social constructs, the various characteristics and genders can change over time, and/or across various cultures (21, 22). Because of its societal nature, the treatment of individuals not adhering to gender norms have faced varying degrees of penalty depending upon the time period, geographic regions, and culture and politics (3, 21, 41). Attempting to review all permutations of all characteristics which are associated with gender is beyond the scope of this review. However, any individuals having characteristics, traits, or gender identities not aligned with the societal norms for their biological sex may be considered some degree of gender non-conforming and may be subjected to similar castigation as transgender individuals.

The term transgender is relatively modern and predated by terms such as transvestite or transsexual prior to the 20th century (42). However in the 1960s, the term transgenderism became more common as it was noted that sexuality was not necessarily related to being transgender (43). While transgender may not perfectly capture the range of possible gender identities, it expresses the idea of sex and gender being unaligned (17, 44). Thus, cisgender was coined to describe an individual whose sex and gender are the same (44). Discussion of cis and trans gender individuals, and their rights in society may seem to be a modern issue, however, these discussions date back, at least, to the 19th century (45). Historical studies from these times document individuals who may be considered transgender by modern understanding (45–47). Some historians and activists argue that examples of people assuming unconventional gender roles or breaking with gender norms have existed throughout history (46, 47). However, it is nearly impossible to determine the gender identities of historical figures, as these individuals likely had no concept of gender identity in the modern context; additionally, there were harsh consequences for those who deviated from societal gender norms at various points in history (45–47). Thus, the current review will only focus on modern issues of gender identity as they relate to participation in sports and athletics.

### 2.2. Sports and gender classifications

When discussing sex, gender, and participation in sports and athletics, the main point of contention seems to be who should or should not be allowed to participate. The International Olympic Charter states that “The practice of sport is a human right. Every individual must have the possibility of practicing sport, without discrimination of any kind…” (48). If taken on its face, this Olympic principle indicates that trans individuals should have no restrictions on access to sports and athletics, and that discrimination violates that human right. The International Olympic Committee follows this principle in their most recent framework on fairness, however leaves the final decision on inclusion of transgender individuals to the various international federations on the basis of fairness and safety (49). This departure from the IOC consensus meeting on sex reassignment and hyperandrogenism acknowledged that one set of regulations did not account for the variability in different sport and athletic requirements, thus international federations would be best placed to make final determinations (49–51). The IOC stressed the importance of an evidence-based approach to assessing under what circumstances trans individuals should be restricted from participation (49, 50). Many of the international federations do choose to restrict participation in their respective athletic events on the basis of either trans status or, based on the blood concentration of hormones for trans individuals (8, 11, 52). It is common for sport/athletic federations to cite certain scientific facts and studies to justify their provisions (8, 11, 52). However, while it is true that some average differences can be measured between cis and trans individuals, these can vary across different studies and different physiologic traits (15, 53–56). While the sport/athletic federations provisions focus on mean differences between cis and trans individuals, they tend to ignore differences amongst a cis only population that lead to advantages, disadvantages, or confer no advantage in athletics and sport performance (11, 38, 40, 57–59). The total number of cis individuals that are naturally advantaged or disadvantaged would likely exceed the total number of transgender individuals wishing to compete based on their low proportion of the total population (60). Hence, the exclusion of the transgender individuals from sports and athletics based solely on concerns of inequity or injury risk, may be a solution in search of a problem.

## 3. Physiologic sex differences and their effects on performance among cisgender individuals

To discuss differences in exercise and performance among cis and trans gender individuals, there must first be an understanding of biologic sex differences, and the concern over what traits are lost or retained following an individual's transition. These differences arise from a combination of developmental, morphological, and hormonal differences between males and females (61). Some of these differences occur as a result of growth and development in puberty, while others are a result of persistent endogenous hormone concentrations (62–66).

### 3.1. General differences from development through adulthood in cisgender individuals

Sex differences are not as pronounced among younger males and females but emerge among adult males and females (63–66). Prior to puberty, young males and females have similar aerobic capacity, strength, body composition, and overall athletic performance (63, 65, 66). However, studies do find slight advantages in anaerobic and strength performance, such as running and throwing, among young males compared to females, while young females have similarly slight advantages in flexibility (67, 68). It is uncertain to what extent this is a result of physiologic differences, rates of maturation, or sociologic differences, as young males are more likely to spend time engaged in physical activity compared to young females which may also lead to differences in performance (69, 70).

The magnitude of sex differences increases as individuals mature (65). Adolescent and adult males and females show average differences in aerobic capacity, body composition, and strength (61). These are interrelated but will be addressed individually. As children grow and move through puberty, both sexes have increases in body size, although to a greater extent among males, leading to greater male average mass and height (62, 71). During puberty, differences in body composition become apparent, with males having lower average body fat, and higher average muscle mass compared to age matched females (64, 72, 73). This difference in body composition leads to increasing strength and aerobic capacity to a greater extent among males than females (64, 74). Due to a greater proportion of lean mass to total body mass, males on average are able to produce greater force for their body size (75–77). This can lead to advantages in sports where strength and power are valued (56, 78). Despite average differences, it is worth noting that overlap exists between the normal upper and lower ranges of males and females (62). The differences in size and composition of the body do not only relate to strength and power, but also to aerobic focused athletics.

Prepubertal males and females have similar aerobic capacities relative to body mass (63, 79). However, post-pubertal males show greater aerobic capacity compared to females due to changes such as increased hemoglobin and leaner body composition (64, 73, 74, 80, 81). The average sex differences in body composition favors higher relative aerobic capacity among males whose greater muscle mass and lower fat mass allows for greater uptake of oxygen per kilogram of total body mass during physical activity (79, 82, 83). Additionally, males will surpass females in left ventricular end diastolic volume and ventricular wall thickness, allowing greater cardiac output at similar heart rates (84). This is aided by the greater average hematocrit/hemoglobin among males compared to females (85–87). From birth to the onset of puberty, hematocrit/hemoglobin values are generally similar, but during maturation, males have a greater increase in hematocrit/hemoglobin compared to age matched females, although there is still overlap in normal ranges between these groups (85, 87). The combination of greater proportion of lean mass, heart size, hemoglobin leading to greater aerobic capacity leads to generally faster race times in many aerobically focused events (88–91).

**Figure 1:**
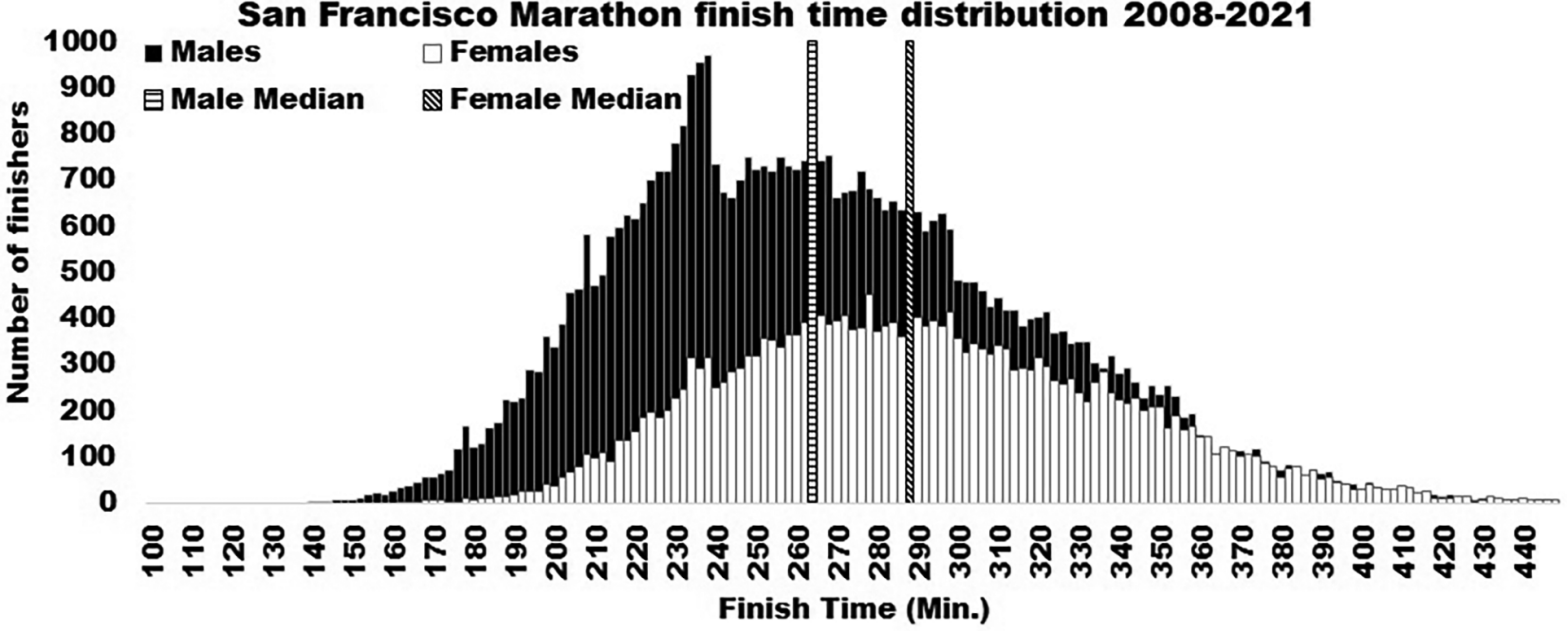
This figure shows the number of males and females finishing times for the San Francisco marathon from 2008 to 2021.

Despite these sex differences that develop during puberty, there are still large areas of overlap in performance, the degree of which is dependent upon the physical demands of the activity (56, 78, 92, 93). An example can be seen in Figure 1, showing the overlap in finish times of the San Francisco Marathon runners 12 years and older (94). The overlap in performance is due to variability in body sizes, individual training and preparation, genetics, proper diet and hydration status, and likely other variables. It is also worth noting that across the history of marathons, including the data from the San Francisco Marathon, fewer total women have participated (94). As more women become involved in sport and improve in training, the gap in performance may narrow further (95). However, males, on average, seem to enjoy an advantage in many athletic competitions, due largely to the effects of testosterone playing out during pubertal development and through adulthood (36, 56, 66).

### 3.2. Hormonal differences in cisgender individuals from puberty and beyond

Many of the sex differences that become apparent during development are a result of hormonal differences that occur during puberty and persist beyond (66). In general, males will have higher testosterone concentrations than females, but lower estrogen concentrations relative to females (36, 37, 39, 96). However, the average difference in estrogen concentrations between males and females is less pronounced than the difference in testosterone (36, 37, 39, 96). Thus, it is important to understand how these different sex hormones contribute to physiologic factors associated with physical performance. Testosterone is known as an anabolic and androgenic hormone while estrogen is more commonly associated with soft tissue and bone health (36, 97, 98). Therefore, these will be discussed beyond the roles they play in development during puberty, to the characteristics they maintain in adults.

The effects of estrogen on performance have been studied predominantly in female athletes. How, or if, estrogen affects performance is not entirely understood due to changes in hormonal concentrations through the menstrual cycle, alterations of estrogen and progesterone resulting from oral contraceptive use, and changes in hormonal concentrations occurring around menopause (37, 99–101). Despite these limitations, several investigations have examined how performance may be influenced at different phases of the menstrual cycle (102, 103). While estrogen is associated with changes in cardiovascular function, respiratory function, thermoregulation, and substrate metabolism, the impact of the estrogen variations across the menstrual cycle have not shown consistent advantages or disadvantages in sports performance (92, 104). However, there are changes in estrogen before, during, and after athletic competitions in both males and females, thus future research may find that these changes are influencing performance (37, 96).

By contrast, testosterone, and its influence on athletic performance, has been intensely studied in both males and females. This is in part due to the abuse of testosterone, and/or closely related compounds, as ergogenic aids (105–107). The difference in testosterone concentration between sexes are wider than those seen with estrogen and are unlikely to overlap between sex groups with males having as much as 20 × the testosterone of females (36–38, 40). However, there is interindividual variation within sexes, with some males or females having as much as double the testosterone of their peers (36–38, 40). Despite the difference in hormone concentration between sexes, testosterone promotes muscle growth and strength in both males and females (106, 108–111). Even within a single group, such as cis men or cis women, serum testosterone concentrations seem to relate to enhanced muscle mass, strength, aerobic endurance, and possibly psychological advantages (108, 112–114). Although, the degree to which this influences performance is questionable and affected by the total difference in hormone concentrations and in sport specific demands (114–116). This issue can become contentious due to the variability in endogenous testosterone levels among athletes, even within a single cisgender category (113, 117). For example, there may be 2–3 × more or less testosterone within either elite male or female athletes and still be within normal ranges (59, 66). On the other hand, cis men with naturally occurring hyperandrogenism are not limited in their ability to compete while a cis woman with hyperandrogenism would be (118–120). This is despite the fact that this, as well as many other genetic/biologic factors, are beyond the control of the individual athletes (118, 121).

## 4. Cis and trans gender performance differences

Thus, there are clearly average differences in performance between males and females, despite an overlap in the average distribution (depending on the sport, age, and skill level). However, transgender individuals, particularly those who have undergone gender affirming hormone therapy and/or gender affirming surgical procedures, have physiologic and potentially morphologic differences from their pre-transition cisgender counterparts (16, 53, 54, 122). Therefore, it is not sufficient to examine sex differences among cis men and women and apply these directly to trans men and women, nor is it sufficient to examine pre to post transition without some cisgender comparisons. Unfortunately, there is a dearth of research on cis and trans gender differences. This lack of data are due to several factors: (1) trans individuals make up only 0.5% of the general population, and likely smaller proportion of highly trained athletes, making this population difficult to recruit and study, (2) highly trained individuals should not be compared to recreationally trained or untrained individuals and few highly trained trans individuals are able to be recruited within a single athletic discipline for a study (3) being trans does not necessitate gender affirming hormone therapy or surgeries, allowing various physiologic and morphologic possibilities amongst the trans community (50). Despite these limitations, attempts have been made to compare cis and trans gender individuals on many parameters of athletic performance, or proxies for these parameters (such as hand-grip strength) (12, 53–55, 122, 123).

In general, studies find that trans individuals, following gender affirming hormone therapy, become more similar to their gender identity (post-transition) cisgender counterparts, or are somewhere between the expected male and female averages (53–55, 122). Certain aspects of pre-transition-sex seem to be less malleable, such as total height and limb length (53, 122). However, there are changes in aerobic capacity, body composition, and muscular strength and endurance (12, 16, 53, 54, 122). Although these parameters may take months or years to complete this transition (12, 53, 54). The time to transition is relevant as (1) earlier in the process of transition, trans individuals may still retain traits more similar to their pre-transition gender, and (2) the effects of transitioning become confounded with the effects of aging and changes in training status (54, 55, 124–126). Due to all the potential pitfalls, a single definitive study of this topic is impossible, however, those which have been attempted can be used to glean better understanding of whether trans individuals' participation in sports and athletics is likely to lead to increased inequity or injury in sports and athletics.

Using a large sample among transgender exercise studies, Roberts et al. showed how the performance of both trans men and trans women on military physical fitness assessments changed over two years of gender affirming hormone therapy (54). Roberts et al. assessed number of push-ups and sit-ups performed as a field assessment of muscular strength and endurance (54). In addition, a 1.5-mile run was used as a field assessment of aerobic capacity (54). It was notable that neither trans men nor trans women aligned perfectly with their cisgender counterparts prior to gender affirming hormone therapy, with trans men performing more pushups and sit-ups (37.4 ± 2.03 and 50.4 ± 1.47) than cis women (32.6 ± 2.12 and 45.6 ± 1.51), and trans women performing fewer pushups than cis men (47.3 ± 1.34 vs. 53.5 ± 1.35) (54). Despite this, these groups were otherwise similar (54). Following two years of gender affirming hormone therapy, trans men showed no differences in pushups or 1.5 mile run time from cis men (56.1 ± 3.05 and 711 ± 34.91sec. vs. 51.5 ± 4.31 and 720 ± 35.71sec.), however they surpassed cis men for number of sit-ups performed (58.3 ± 2.20 vs. 52.4 ± 2.27) (54). For trans women following gender affirming hormone therapy, there were no differences in sit-ups or push-ups performed from cis women (44.8 ± 3.79 and 34.6 ± 4.21 vs. 45.7 ± 3.85 and 32.5 ± 4.31). However, post gender affirming hormone therapy, trans women still surpassed cis women for their 1.5 mile run time (765 ± 39.83 s. vs. 855 ± 40.56 s.), but performed significantly slower than cis men (720 ± 40.56 s.) unlike their pre hormone therapy assessment (54).

This study by Roberts et al. has also been used to support restricting trans individuals from participation in sport and athletic competitions (127, 128). As described above, following 2 years of gender affirming hormone therapy, trans women completed their 1.5 mile run slower than cis men, but still faster than cis women, raising potential concerns (54, 127). Fortunately, some of the original research team from the Roberts et al. study continued to follow-up for four years (129). However, the new analysis did not necessarily use the same participants as Roberts et al., having higher sample sizes for earlier years and lower sample sizes as they moved to year four. The follow-up study by Chiccarelli et al. noted that equivalence testing [the Two One-Sided Test (TOST)] should be used in addition to standard hypothesis testing to compare transgender individuals with their cisgender counterparts. The study also made comparisons of trans individuals' own pre-transition and post-transition performance as percentiles for men or women (129). Using these approaches, the researchers showed that trans women performance on the 1.5 mile run was not statistically different from cis women times following two years of gender affirming hormone therapy and remained equivalent to cis women out to year four (874 ± 133 s vs. 876 ± 111 s.) (129). Furthermore, the TOST analysis showed that the trans womens' female percentile scores were equivalent to their pre-transition male percentile scores (129). These findings are similar to a study by Harper et al. which showed similar age adjusted running scores pre and post transition (55). However, Chiccarelli et al. they did find that the pushup test for trans women did not decline enough to be equivalent to their pre-transition percentile ranking, and the number of push-ups performed were still greater than those performed by cis women (35.3 ± 7 vs. 30 ± 10) (129). Trans men also continually performed more like cis men and significantly better than cis women over the four years, and it is worth noting that by year four of follow-up, trans men were out performing cis men on both pushups and sit-ups (129). Based on these data, trans men and trans women continually performed more similarly to their affirmed cisgender performance averages, and approach their own pre-transition percentile scores over the four years of gender affirming hormone therapy (129). Although these findings seem to indicate that trans individuals may eventually reach equivalence with their cisgender counterparts, it should also be noted that the participant pool diminished over the four years of follow-up, increasing the risk of self-selection bias and increasing risk of potential error (14, 129).

A study by Jenkins et al. were able to account for more granular fitness parameters among trans women than those used by Roberts et al. and Chiccarelli et al., however this came at the cost of a small total sample of trans women of various ages and physical activity levels (53). To best account for these differences, trans individuals were matched with cis men and women of matched age and activity levels. All the trans women had been using gender affirming hormone therapy for at least two years allowing the researchers to compare many of their fitness attributes to cis men and women. Trans women indeed retained body mass and height significantly greater than cis women, and similar to cis men (53). However, their body composition (25.17 ± 8.57% body fat) was between that of cis men (17.12 ± 5.22%) and cis women (32.98 ± 9.06%), and not statistically different from either (53). Trans women were also shown to have higher grip-strength than cis women (93.0 ± 14.0 kg vs. 63.9 ± 9.5 kg) with no difference from cis men (112.7 ± 23.2 kg) (35). These findings do align with other studies that find strength and muscle mass do not adapt as quickly to gender affirming hormone therapy as cardiorespiratory parameters (14, 16, 54, 127, 129). However, on more performance related tests of: vertical jump height, pushups, and maximal aerobic capacity, trans women performed similarly to, or less than, cis women (33.98 ± 4.34 cm, 17.83 ± 5.46, and 29.43 ± 9.41 ml/kg/min compared to 34.24 ± 7.84 cm, 19.67 ± 11.64, and 30.43 ± 9.15 ml/kg/min), and significantly less than cis men (47.34 ± 6.43 cm, 48.0 ± 5.37, and 41.15 ± 13.77 ml/kg/min) (53). With such a small sample, this should not be overly interpreted, although it does seem to align with findings from Roberts et al. and Chiccarelli et al.

A slightly larger study by Alvares et al. also examined body compositional differences between cis men and women, and trans women (12). The study by Alvares et al., unlike data from Jenkins et al., showed a significantly higher percent body fat among trans women compared to cis men (29.5 ± 1.47% vs. 20.2 ± 1.52%), with no differences between trans women and cis women (32.9 ± 1.58) (12). Despite no differences in percent body fat, trans women still showed greater lean mass than cis women (30.7 ± 0.85 kg lean mass vs. 21.9 ± 0.67 kg lean mass) (12). Unlike the study by Jenkins et al., Alvares et al. measured a much smaller difference in grip strength between cis women and trans women (29.7 ± 1.0 kg vs. 35.2 ± 1.39 kg), with both being significantly lower than cis men (48.4 ± 1.79 kg) (12). Both studies seem to agree that relative aerobic capacity of trans women is decreased to levels similar to those of cis women (33.5 ± 1.21 ml/kg/min vs. 35.7 ± 1.30 ml/kg/min (12, 53). Alvares goes further and quantifies hemoglobin levels showing that trans women have similar hemoglobin to cis women (14.0 ± 0.15 g/dl vs. 13.8 ± 0.17 g/dl) and less than cis men (15.3 ± 0.29 g/dl) (12). These same findings for hemoglobin changes have been shown in other gender affirming hormone therapy treatments, and the subsequent changes in running performance are even shown among trans athletes (13, 55, 130).

Unfortunately, few studies are able to examine trans athletes due to the small population. A study by Harper et al. examined runners' race times across transition from men to women to determine how transitioning affected their performance (55). The study followed nine athletes over an average of 7 ± 1.9 years of transition (range of years of transition = 1–29). Harper found that there was a decrease in performance measured by race times (55). To account for the aging effect on running performance, Harper et al. used age grading, comparing run times to a standard run time accounting for race distance and sex. The age grades were on average, unchanged following transition (68.7% vs. 68.5%), suggesting that athletes as trans women did not enjoy an advantage compared to their pre-transition abilities. In other words, as trans women, they were performing at a similar competitive level, compared to other cis women, as they had as cis men compared to other cis men (55).

While data are still scarce, the limited information available does not suggest that trans men and trans women have much, if any, athletic advantage post-transition. Indeed, in most cases they perform more similarly to those matching their gender identity, or somewhere between cis men and women (12, 15, 53–55). If these individuals are performing somewhere between cis men and women on some performance parameters, does it pose a meaningful risk of inequity in sport or risk of athletic injuries, or are concerns for these problems misplacing blame to cover discrimination?.

## 5. Misplacing blame

**Figure 2:**
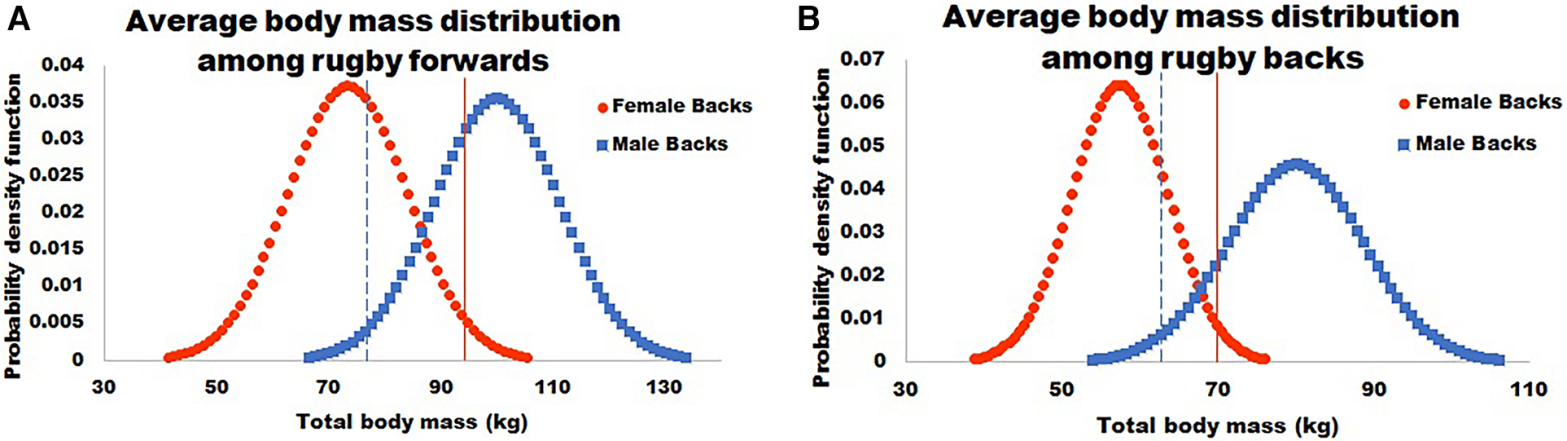
This figure shows the average body mass distribution among male (squares) and female (circles) rugby players. It also notes 2 standard deviations to the high end for females (solid line) and to the low end for males (dashed line). Data calculated based on values reported in Ramos-Álvarez et al. 2021 (12).

**Figure 3:**
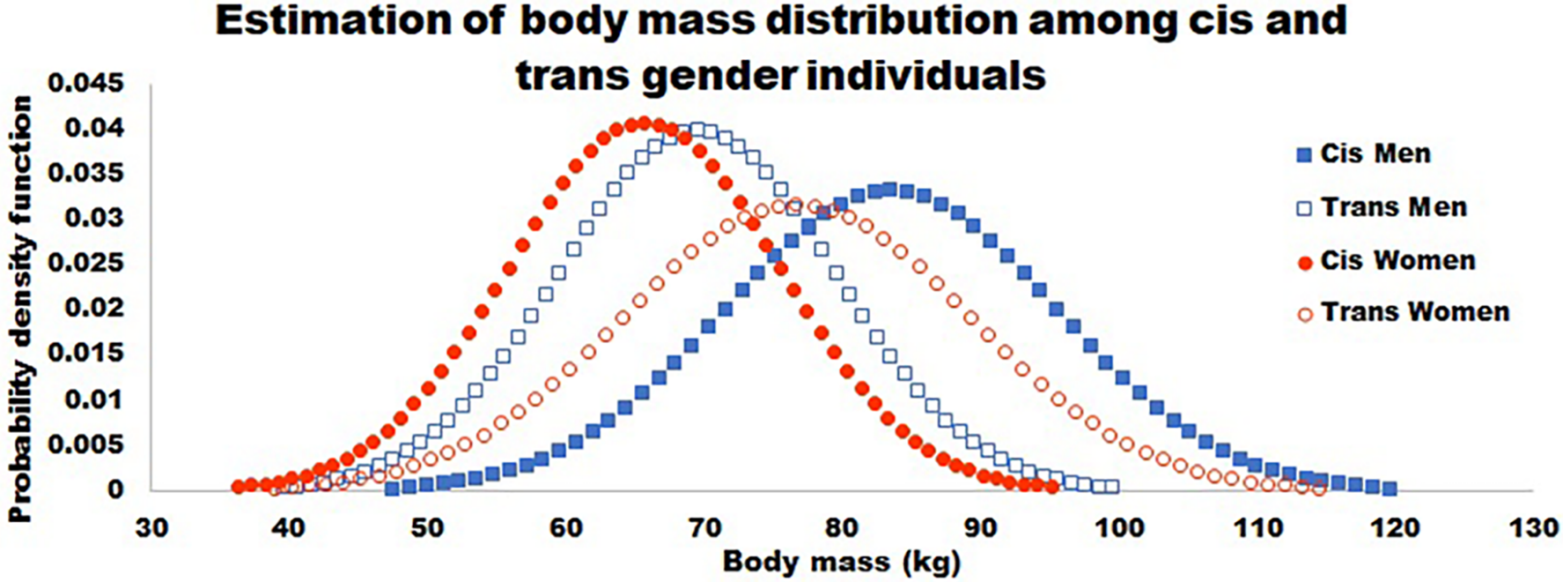
This figure shows an estimation of body mass distributions before gender affirming hormone therapy based on values reported by Roberts et al. 2020. Men and women are shown in squares and circles respectively, with cis and trans being denoted by closed or open symbols. As Roberts showed, there are not differences between the cisgender individuals and those who transitioned from that gender. However, there are large areas over overlap estimated among these populations even before gender affirming treatments.

As reviewed above, there are in fact sex differences on average performance values between males and females. However, too often, these differences are used as evidence to restrict trans individuals from sports and athletics (11, 52). The average differences observed between males and females are cited as problems leading to inequality and elevated risk in sports and athletics despite evidence that trans individuals do not match their pre-transition cisgender counterparts (11, 131). However, if these average differences lead to inequity or injury, restricting trans individuals from these sports and athletics may not be the best solution. This can be approached similar to a public health issue, assessing what important factors influence outcomes, and what effect would come from altering individual factors (i.e., the population attributable risk). For example, Transgender Guidelines by World Rugby state concern for the larger average mass of males relative to females, and how this influences the force of impact (i.e.,: mass × acceleration) (11). However, when looking at a sample distribution of players, >300 males sampled fall below the 2nd percentile of average male body mass and >300 females sampled are above the 98th percentile of average female body mass (11). If being too large or too small were a critical concern for rugby injuries, more injuries may be prevented by restricting those >600 players who fell far outside the average player mass than banning trans athletes. If average body mass values and their standard deviations for elite rugby players were used to generate a normal distribution of mass, there would still be areas of overlap among females and males. This can be seen in Figure 2 in which distributions were calculated from data reported by Ramos-Álvarez et al. using the following equation: $f(x;,\mu ,\sigma )=\frac{1}{\sqrt{2\pi \sigma }}e^{-\left(\frac{{\left(x-\mu \right)}^{2}}{2\sigma^{2}}\right)}$ (57). Injury in sport is a serious problem, however a ban on trans individuals does not solve this problem because there is already normal variation among cisgender individuals (53, 54). Using average pre-transition body sizes reported by Roberts et al., an estimation of the body mass distributions among cis and transgender men and women were estimated (assuming a normal distribution) showing large areas of overlap, even before gender affirming hormone therapy, as shown in Figure 3 (54). Perhaps in this case, if injuries are a primary concern, rugby should have weight classes similar to other sports where body mass is an important parameter rather than a ban on trans athletes.

Another example of misplaced blame would be concerns that trans women have too much testosterone or that trans men are gaining an unfairly advantage by taking testosterone (8, 11, 56, 127). Herein lies the myth that cis men, not on gender affirming hormone therapy, will claim to be a trans women to win at female sporting events. However, trans individuals use gender affirming hormone therapy to better match their gender identity, not to gain unfair sporting advantages (132). While it is true that certain morphological changes that occur during puberty may be irreversible, trans individuals on gender affirming hormone therapy clearly do not retain the same physiologic parameters as their pre-transition counterparts (12, 15, 16, 54–56, 129). It is unclear to what extent, or for how long, any hormone mediated advantages may persist once a trans individual begins regular gender affirming hormone therapy (12, 15, 53, 56, 129). It has been shown that parameters affecting aerobic performance transition more quickly than those affecting strength performance (16, 127, 129). However, excluding trans individuals does not prevent cases of athletes having hormonal advantages. The World Anti-Doping Agency (WADA) found 93 of the 4,422 athletes tested (2.1%) to have “adverse analytical finding” for steroid use (105). WADA does not report how many of those athletes were cis or trans, however, as transgender individuals are underrepresented in athletics, it is likely that these are cis athletes (60). Besides use of exogenous hormones, cisgender individuals naturally vary in their hormonal profiles (40, 59, 66, 133). Thus, restricting trans individuals is unlikely to prevent issues of ergogenic hormonal advantages in sports.

Finally, it is well known that within sports and athletics, competitive advantage is in large part influenced by genetic predisposition (121, 134, 135). It is accepted that some individuals are born with natural advantages, however, the suggestion that trans individuals may enjoy some advantage in certain cases is regarded as unacceptable. Yet there does not seem to be a domination of sports by trans athletes if their advantage is so great. When examining issues that allegedly arise by trans athletes' participation in sports and athletics, the solutions are more driven by a political/cultural divide rather than an honest attempt to actually mitigate inequities or risk of injuries that are occurring (1, 136).

## 6. Limitations and future directions

There are several limitations and future directions in this topic area. Many others have also noted that there is a need to study individuals who are both trans and elite athletes. Currently, the limited knowledge of athletic performance among trans men and women are limited to individuals who are either moderately active, recreationally trained, or military trained individuals (53–55, 129). Due to trans individuals making up such a small proportion of the total population, and their disproportionate rates of discrimination, recruitment of highly trained trans individuals is one of the greatest difficulties in this area of research (2, 7). In addition to difficulty in recruitment, the performance difference between males and females across different sports are not uniform (56, 92, 137). Individual components of fitness or task proficiency do not always translate to sport and athletic performance. Thus, fitness assessments do not account for training and skill at sports/athletics which may influence success as much as physiologic potential. Finally, there is the problem of neither sex nor gender being true binaries. This makes it nearly impossible to make comparisons among these individuals when classified into superimposed categories. As an example, even individuals who are cisgender, may have differences in sexual development, or have sexual aneuploidy, but would be simply classified based on their gender identity unless they were outed. Trans individuals, on the other hand, may have had various surgical procedures or take different gender affirming hormone therapy to better match assumed sex standards. All of these factors would need to be accounted for to truly understand what extent these individuals vary in sport and athletic performance. Future studies will be essential to gain a more comprehensive understanding of this population, and conclude whether and to what extent sex differences are retained post-transition.

## 7. Conclusion

Individuals should not have to make a choice between being their authentic selves or being athletes (138). While trans athletes competing in various sports and athletic events raises interesting considerations of how certain morphologic and physiologic factors affect performance, these questions are not exclusive to trans individuals. There are wide variations within cisgender populations, even when excluding individuals with differences in sexual development (121, 139). It is expected that about 2.3% of a normally distributed population is likely to fall above two standard deviations from a population mean. These exceptional individuals may be those who are gifted and excel at some sport or athletic performance (121, 135, 140). In contrast only 0.5%–0.6% of the population identify as trans (60). There is no concern for restricting individuals who are exceptionally large or small, those who are genetically gifted, or those with differing hormone concentrations or muscle mass, so long as their gender and biologic sex align (120, 121). The disproportionate focus on the relatively small portion of the population who are trans seems based on the belief that cis men, who cannot succeed in sports among other cis men, would choose to misidentify as trans women to gain an advantage in sports against cis women. However, there are no legitimate cases of this occurring. An individual's sex does not determine their success or failure at any athletic event despite the high level of competition. This can be demonstrated when looking at not average outcomes, but the level of overlap among outcomes. The exclusion of trans individuals also insults the skill and athleticism of both cis and trans athletes. While sex differences do develop following puberty, many of the sex differences are reduced, if not erased, over time by gender affirming hormone therapy. Finally, if it is found that trans individuals have advantages in certain athletic events or sports; in those cases, there will still be a question of whether this should be considered unfair, or accepted as another instance of naturally occurring variability seen in athletes already participating in these events.
